# Two cases of emphysematous cystitis with urinary bladder rupture

**DOI:** 10.1093/omcr/omag018

**Published:** 2026-03-23

**Authors:** Xuan Thai Ngo, Duc Huy Vu, Nguyen Xuong Duong, Phan Nhat Duy Le, Duc Minh Pham, Ryan W Dobbs, Hoai Tam Ly, Hoai Phan Nguyen, Huu Thuan Le, Tien Dat Hoang, Minh Sam Thai, Tuan Thanh Nguyen

**Affiliations:** University of Medicine and Pharmacy at Ho Chi Minh City, Department of Urology, 217 Hong Bang Street, Ward 11, District 5, Ho Chi Minh City, Vietnam; Cho Ray Hospital, Department of Urology, 201B Nguyen Chi Thanh, Ward 12, District 5, Ho Chi Minh City, Vietnam; Cho Ray Hospital, Department of Urology, 201B Nguyen Chi Thanh, Ward 12, District 5, Ho Chi Minh City, Vietnam; Cho Ray Hospital, Department of Urology, 201B Nguyen Chi Thanh, Ward 12, District 5, Ho Chi Minh City, Vietnam; University of Medicine and Pharmacy at Ho Chi Minh City, Department of Urology, 217 Hong Bang Street, Ward 11, District 5, Ho Chi Minh City, Vietnam; University of Medicine and Pharmacy at Ho Chi Minh City, Department of Urology, 217 Hong Bang Street, Ward 11, District 5, Ho Chi Minh City, Vietnam; Cho Ray Hospital, Department of Urology, 201B Nguyen Chi Thanh, Ward 12, District 5, Ho Chi Minh City, Vietnam; Cook County Health and Hospitals System, Department of Urology, 1901 W. Harrison St. Chicago, IL 60612, United States; Cho Ray Hospital, Department of Urology, 201B Nguyen Chi Thanh, Ward 12, District 5, Ho Chi Minh City, Vietnam; Cho Ray Hospital, Department of Urology, 201B Nguyen Chi Thanh, Ward 12, District 5, Ho Chi Minh City, Vietnam; Cho Ray Hospital, Department of Urology, 201B Nguyen Chi Thanh, Ward 12, District 5, Ho Chi Minh City, Vietnam; Cho Ray Hospital, Department of Urology, 201B Nguyen Chi Thanh, Ward 12, District 5, Ho Chi Minh City, Vietnam; University of Medicine and Pharmacy at Ho Chi Minh City, Department of Urology, 217 Hong Bang Street, Ward 11, District 5, Ho Chi Minh City, Vietnam; Cho Ray Hospital, Department of Urology, 201B Nguyen Chi Thanh, Ward 12, District 5, Ho Chi Minh City, Vietnam; University of Medicine and Pharmacy at Ho Chi Minh City, Department of Urology, 217 Hong Bang Street, Ward 11, District 5, Ho Chi Minh City, Vietnam; Cho Ray Hospital, Department of Urology, 201B Nguyen Chi Thanh, Ward 12, District 5, Ho Chi Minh City, Vietnam; University of California Irvine, Department of Urology, 260 Aldrich Hall, Irvine, CA 92697, United States

**Keywords:** emphysematous cystitis, spontaneous urinary bladder rupture, urinary tract infection

## Abstract

Spontaneous urinary bladder rupture due to emphysematous cystitis is a rare and life-threatening complication, which often presents with atypical symptoms leading to delayed diagnosis and treatment. We present two cases of middle-aged Vietnamese females who initially presented with emphysematous cystitis and whose clinical course was complicated by spontaneous urinary bladder rupture as well as a literature review of this uncommon life-threatening urologic complication. Urinary bladder rupture is an uncommon condition which may occur from instrumentation, infection or idiopathic etiologies and requires a high degree of clinical suspicion for diagnosis. Our cases highlight the need for multidisciplinary coordination to manage underlying diseases and in-depth timely evaluation to detect and address potential complications that may arise.

## Introduction

Emphysematous cystitis (EC) is a necrotizing infection of the urinary bladder caused by gas-forming organisms. While the clinical spectrum varies from asymptomatic bacteriuria to fulminant septic shock, spontaneous bladder rupture represents a catastrophic and exceptionally rare complication [1, 2]. This condition demands urgent surgical intervention but is frequently associated with diagnostic delays and high mortality due to its non-specific presentation [3]. Therefor, we report two cases of EC complicated by spontaneous bladder rupture to highlight the critical need for early recognition and prompt management.

## Case presentation

### Case one

**Figure 1:**
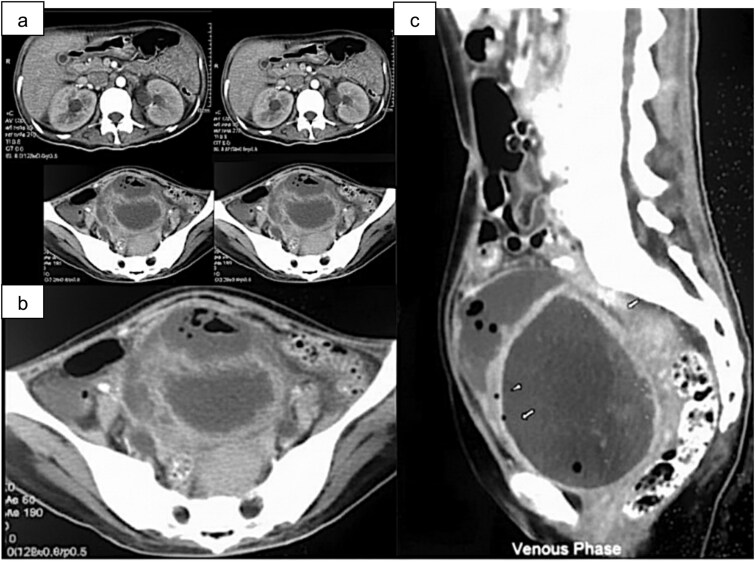
Contrast-enhanced abdominal CT. (a) Bilateral hydronephrosis. (b) Prevesical gas-fluid collection (short arrow). (c) Distended bladder exhibiting intramural (arrowhead) and intraluminal gas (long arrow).

A 47-year-old woman with a history of type 2 diabetes mellitus, presented to the hospital with lower urinary tract symptoms including frequency, hesitancy, and hematuria. On examination, the abdomen was soft, and the hypogastric region was tender. Initial workup was notable for a serum creatinine of 1.81 mg/dl (0.7–1.5), BUN 39 mg/dl (7–20), white blood cell count 8.8 G/l (4–11), hemoglobin 82 g/l (120–180), platelets 261 G/l (150–400), C-reactive protein 103 mg/l (<5), serum protein 5.3 mg/dl (6–8), serum albumin 2.3 mg/dl (3.5–5.5), glycemia 385 mg/dl (70–110), HbA1C 13.8% (< 6.5) and serum ketones 20 mg/dl (negative). Her urine analysis was indicative of a urinary tract infection with a white blood cell count of 500/μl and red blood cell of 200/μl and negative for nitrite. A urine culture was obtained and grew positive for Klebsiella pneumonia. Ultrasound recorded bilateral hydronephrosis, a decrease in urine volume and elevated post-voiding residual. Computed tomography (CT) of the abdomen showed a distended and air-containing bladder and air-fluid level in the pre-vesical space (Fig. 1). This anterior bladder wall fluid collection was consistent with a diagnosis of emphysematous cystitis.

Initial management included intravenous meropenem, insulin, fluid resuscitation, and urethral catheterization for bladder decompression. Due to persistent symptoms on day 4, repeat ultrasound revealed a 4 × 4.4 cm echogenic mass on the anterior bladder wall with calcifications. A peri-vesical abscess was diagnosed, necessitating surgical intervention. Intraoperatively, a 4 × 5 cm abscess and significant bladder wall thickening (1.5 cm) were observed. Crucially, a 2 mm extraperitoneal perforation was identified at the bladder dome. The necrotic edges were debrided, and the defect was closed with interrupted 2–0 Vicryl sutures. Both suprapubic and urethral catheters were placed. Histopathology confirmed chronic inflammation. The patient recovered well and was discharged on postoperative day 5. At one-month follow-up, she remained asymptomatic, and a control CT scan confirmed complete resolution of peri-vesical air and fluid.

### Case two

A 59-year-old female with type 2 diabetes, hypertension, and Cushing syndrome presented with pneumaturia, fever, and suprapubic pain of 3 days’ duration. Admission status was notable for septic shock requiring vasopressors and oxygen support. Significant laboratory findings included leukocytosis (21 G/l), thrombocytopenia (2 G/l), anemia (Hb 85 g/l), glycemia 71 mg/dl, HbA_1_C 12.1%, serum ketones 10 mg/dl, elevated CRP (243 mg/l), ketosis, pyuria, and hematuria. Initial blood and urine cultures were negative.

**Figure 2:**
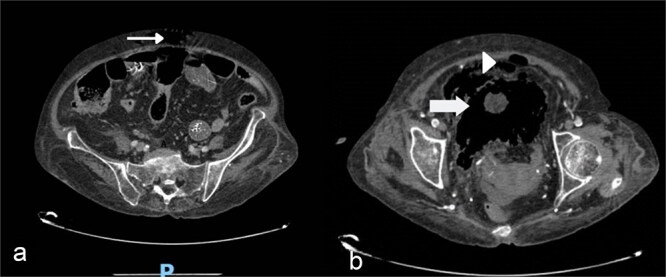
(a) Gas in the abdomen (small arrow), (b) gas spreading in the bladder wall (large arrow) and gas in the prevesical space (arrowhead).

**Figure 3:**
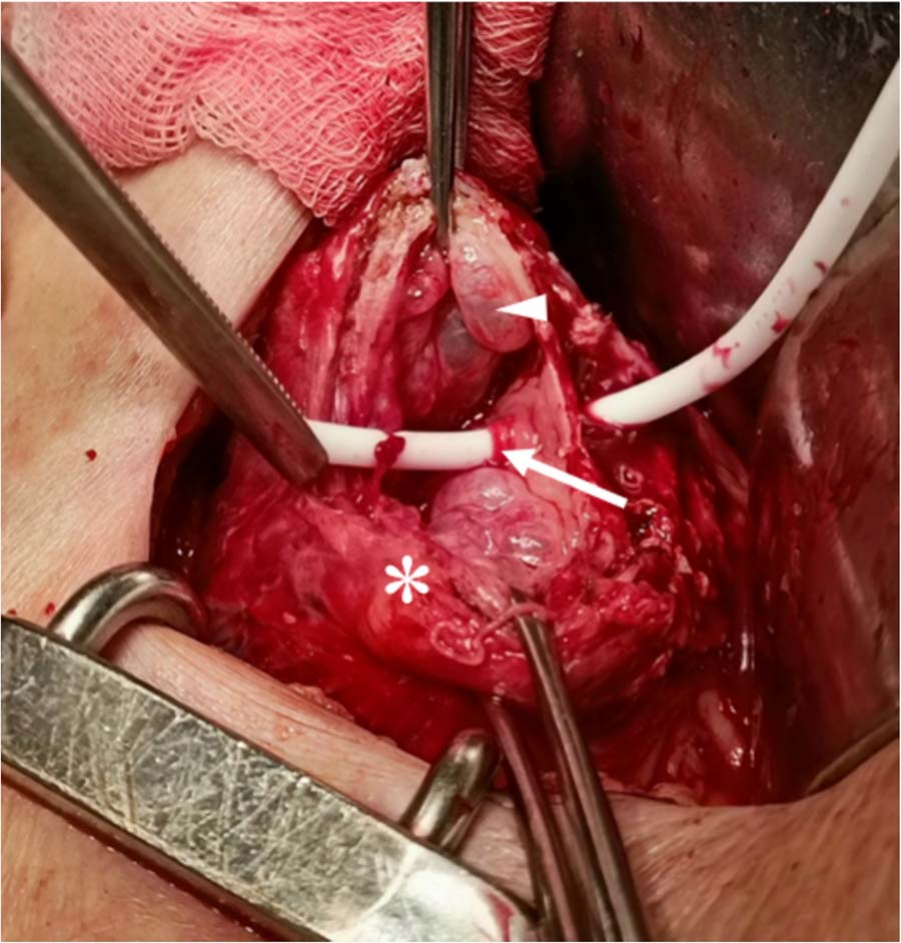
Intraoperative view. Perforation site marked by Nelaton catheter (arrow). Thickened, friable bladder wall (asterisk) and intramural bulla (arrowhead).

Computed tomography (CT) revealed diffuse intramural bladder gas with extension into the prevesical space and abdominal wall soft tissue (Fig. 2), suggesting emphysematous cystitis complicated by perforation. Emergent laparotomy was performed under broad-spectrum antibiotic coverage (imipenem and levofloxacin). Intraoperatively, a right-anterior perivesical abscess and a 5 mm perforation at the bladder dome were identified (Fig. 3). Following debridement of necrotic edges, the defect was repaired in a single layer with 1–0 Vicryl suprapubic and urethral catheters were placed. Intraoperative cultures yielded ESBL-producing *Escherichia coli* sensitive to imipenem. Histopathology confirmed acute inflammation.

On postoperative day 4, she developed acute respiratory failure due to pneumonia. Over the next 2 days, her family refused to continue treatment because of extremely worsening symptoms.

## Discussion

EC is a relatively rare condition. Since the first reported case in 1800s, there have been approximately 237 reported cases in the literature [2, 3]. The disease most commonly affects middle-aged women with diabetes, patients with chronic urinary tract infections, long-term indwelling catheters, lower urinary tract obstruction, neurogenic bladder disorders, and immunosuppression, which are risk factors for gas-forming infections [4, 5]. Risk factors of poor prognosis for gas-forming infections are hemodynamic instability, respiration failure, shock, thrombocytopenia, confusion [6]. In our reported cases, both patients had redisposing factors: female gender, middle age, and poorly controlled blood sugar with diabetes mellitus.

The clinical features of gas-forming bladder infection vary widely from being asymptomatic to septicemia [3]. The most common symptoms include abdominal pain, accounting for 80% of cases, and gross hematuria in 60% of cases [7, 8]. A few cases may experience pneumaturia. Although this symptom is characteristic of gas-forming bladder infection, it is often overlooked or not documented. Passage of gas during urination occurs in 70% of cases with an indwelling bladder catheter [2, 7]. Other acute symptoms of bladder infection, such as frequency, dysuria, and urgency occur in 50% of cases. However, these symptoms are usually nonspecific and mild. Therefore, currently there are no specific clinical features that strongly indicate the presence of EC [2, 9].

A literature review on 135 cases of emphysematous cystitis revealed that the average age of patients was 66 years old, with women accounting for 64% of the cases and a diagnosis of diabetes present in 67% of the cases [3]. The majority of these diagnoses were made based on abdominal X-ray, accounting for approximately 84%, although recently CT scans have become the predominant imaging diagnostic method [3].

Grupper M. et al. found that up to 62.2% of cases of emphysematous cystitis were elderly women with diabetes. Symptoms of urinary tract infection were present in only 53.3% of cases. Common symptoms included abdominal pain in around 65.6% of cases and hematuria in about 82.3% of cases. Abdominal X-ray showed a sensitivity of 97.4%, while abdominal CT scan had the highest sensitivity and served as a specific diagnostic tool [7].

The pathophysiology of emphysematous cystitis remains unclear. Several hypotheses explain the main mechanisms related to gas production inside the bladder leading to emphysematous cystitis, including gas-forming bacterial infections, fermentation of substances such as glucose and proteins in urine, reduced local blood flow resulting in impaired gas absorption due to increased pressure inside the bladder, and lower urinary tract dysfunction or compromised immune defense of the host [9–12]. In this reported case, it is likely that the emphysematous cystitis resulted from a combination of factors such as high glucose levels in the urine, dysregulated bladder innervation, vascular disorders, and compromised immune defenses due to poorly controlled diabetes over an extended period of time.

The majority of isolated pathogens from urine cultures in emphysematous cystitis cases are *E. coli*, accounting for about 58%, followed by *Klebsiella pneumoniae* at a rate of 21% [3]. Other rare causative agents reported include Clostridium spp and Enterobacter spp, Candida ssp, *Pseudomonas aeruginosa*, *Proteus mirabilis*, Group D Streptococcus, Aspergillus fumigatus, and *Staphylococcus aureus* [3].

The treatment of emphysematous cystitis depends on the severity of the disease and includes antibiotics, bladder drainage, blood glucose control, and management of accompanying disorders. Broad-spectrum antibiotics are initially used, and then adjusted based on antibiotic sensitivity.

Conservative treatment may be effective in patients with extraperitoneal bladder rupture cause by *E. Coli* and stable infection status with appropriate antibiotics and bladder drainge, but patients are at high risk of mortality if the localized infection progresses to sepsis and septic shock [13, 14]. Therefore, surgical intervention is necessary for cases that do not respond to medical treatment, severe necrotizing infections, or intraperitoneal bladder rupture. This can have a high success rate and lead to complete recovery in some reports [15, 16]. The rate of surgical intervention is approximately 10% of cases and may involve debridement of necrotic tissue, partial bladder resection, or even total cystectomy [3]. However, when the infection has progressed unfavorably, surgery may also contribute as a significant factor increasing the risk of mortality [17].

In general, the mortality rate of emphysematous cystitis is around 7–9%, with higher rates in cases with severe complications such as bladder rupture due to emphysematous cystitis [3]. However, this is still a rare disease, and further research on more cases is needed to establish an optimal treatment for this complication.

Spontaneous bladder rupture is a rare entity, especially in the context of emphysematous cystitis. Our cases highlight the need for multidisciplinary coordination to manage underlying diseases and in-depth timely investigation to detect and address potential complications that may arise. Further studies are needed to understand the disease process and establish treatment guidelines for emphysematous urinary tract infections.

## Data Availability

The data that support the findings of this study are available from the corresponding author upon reasonable request.
